# Bacterial or Viral: A Case of Central Nervous System Infection in a Young, Immunocompetent Adult

**DOI:** 10.7759/cureus.84801

**Published:** 2025-05-25

**Authors:** Amal Hamieh, Richard Elias, Heba Selim, Joseph Elias

**Affiliations:** 1 Internal Medicine, Al Fujairah Hospital, Emirates Health Services, Al Fujairah, ARE; 2 Neurology, Zagazig University, Zagazig, EGY

**Keywords:** antibiotic use, bacterial meningoencephalitis, culture negative, hsv1 reactivation, pcr multiplex negative

## Abstract

Herpes simplex virus (HSV) is a well-documented cause of encephalitis. However, its occurrence alongside concurrent bacterial infections is less commonly reported. Hence, we present this case report, which aims to trace the etiology of acute encephalitis in our patient, a 22-year-old farm attendant, who initially received intravenous and oral antibiotics that may have distorted the lumbar puncture results. The onset of his illness included typical symptoms such as fever, seizures, and altered mental status. The possibility of bacterial co-infection with HSV-1 viral meningoencephalitis, combined with the early administration of antibiotics in our patient, has inspired this case report to highlight the differential diagnosis concerning the microbiological epidemiology of meningoencephalitis, including concurrent bacterial, viral, and fungal infections.

## Introduction

Herpes simplex virus encephalitis (HSVE) is caused by the human herpesvirus known as herpes simplex virus (HSV) and is the most common fatal sporadic encephalitis in humans.

The annual incidence of HSVE is estimated to be over 4.6 cases per million people, with a 30-day mortality rate of 8.3% following symptom onset [1]. Without adequate treatment, mortality rates can reach as high as 70% [2]. The prevalence of HSV encephalitis and meningitis in adults is estimated at 8% and 4%, respectively. HSVE accounts for approximately 12% of all viral encephalitis cases, with the highest incidence reported in North America and European countries [3].

Bacterial co-infection may complicate the clinical course of HSV encephalitis, potentially leading to worse outcomes, including increased mortality. Therefore, early suspicion and prompt diagnostic screening are essential in prognostic evaluation. Although bacterial and viral co-infections in meningitis are relatively rare (0.8%) [4], close attention to subtle abnormalities in cerebrospinal fluid (CSF) and blood parameters can facilitate early detection and timely treatment.

## Case presentation

A 22-year-old Pakistani male with no known comorbidities, except for a single episode of convulsion one year prior (for which he neither received prophylactic medication nor followed up with a neurologist), presented to the emergency department with complaints of high-grade fever unresponsive to antipyretics (up to 39.4 °C), headache, generalized body aches, mild cervical tenderness, and altered mental status persisting for the past five days.

Five days earlier, he had been admitted to another healthcare facility with similar symptoms of one-day duration, accompanied by a new-onset generalized tonic-clonic seizure. A diagnostic CT scan of the brain was performed during that admission, but the findings were inconclusive. A lumbar puncture was not conducted due to the patient's refusal. Basic blood tests and screening for malaria and dengue were unremarkable. He was started on intravenous levetiracetam 500 mg twice daily and ceftriaxone 2 g twice daily, along with antipyretics, for approximately two days. However, the patient requested discharge against medical advice. At discharge, oral cefuroxime was prescribed, considering a possible febrile illness.

He remained at home for three days before being admitted to our facility with similar symptoms, along with a second generalized tonic-clonic seizure that occurred the day prior to the presentation.

**Figure 1:**
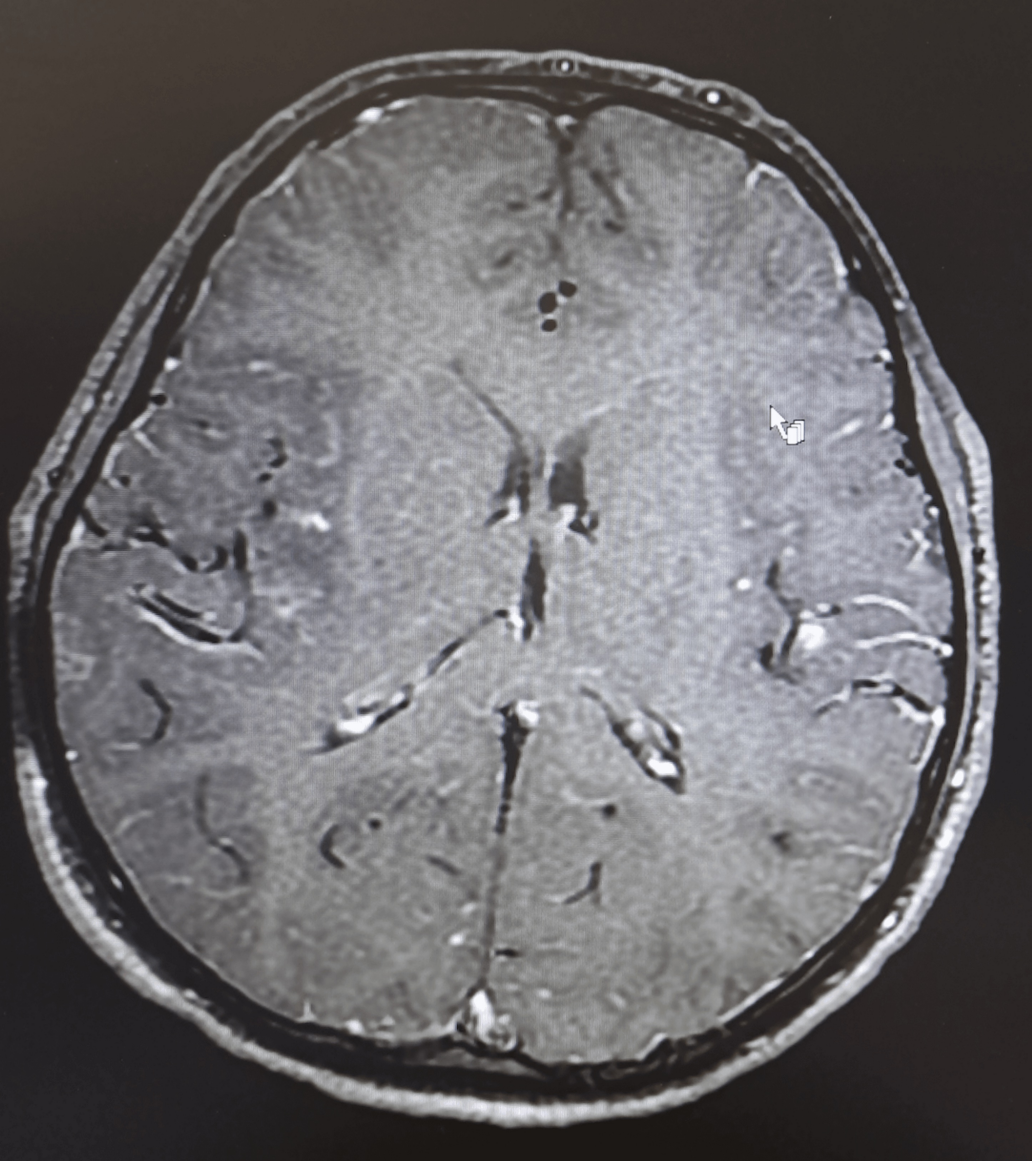
Axial T1-weighted post-contrast MRI of the brain upon admission. It reveals diffuse brain edema and subtle leptomeningeal enhancement over the insular cortex.

**Figure 2:**
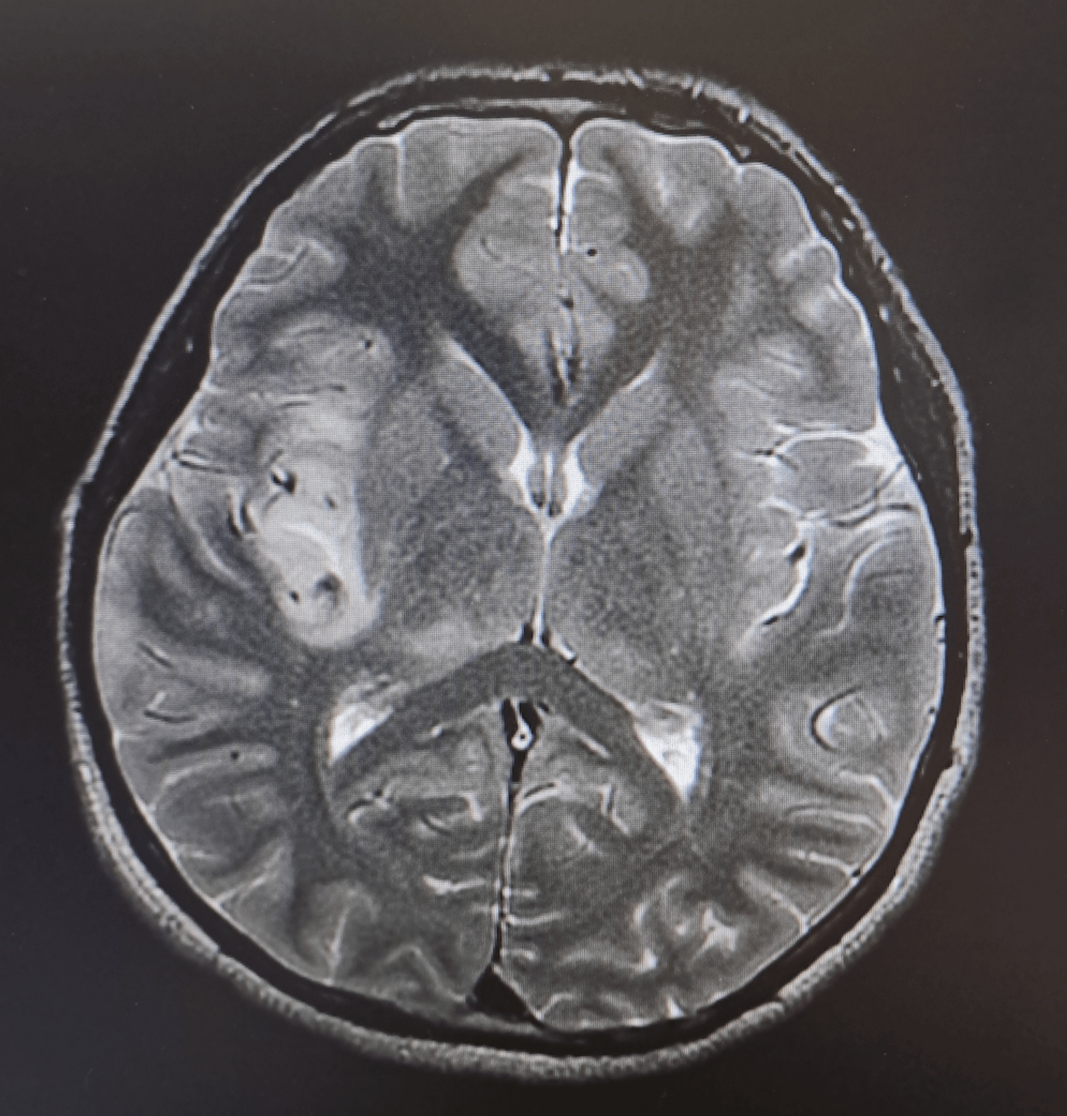
Axial T2-weighted MRI of the brain upon admission. It reveals hyperintensity cortical swelling over the medial temporal insula with few cerebral foci of hemorrhage.

**Figure 3:**
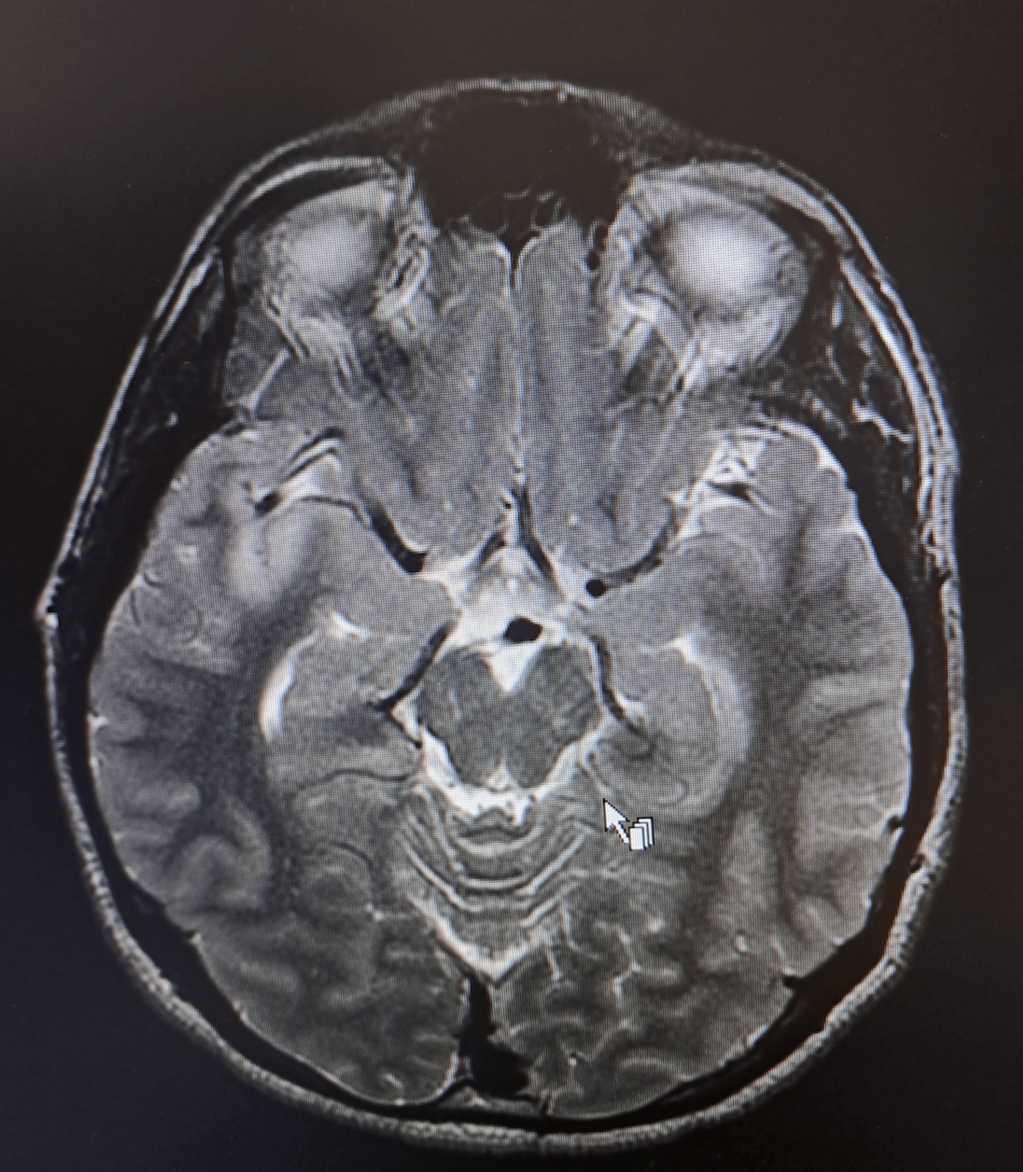
Axial T2-weighted MRI of the brain upon admission. Presence of hyperintense signal with involvement of the right temporal lobe and meningeal enhancement.

**Figure 4:**
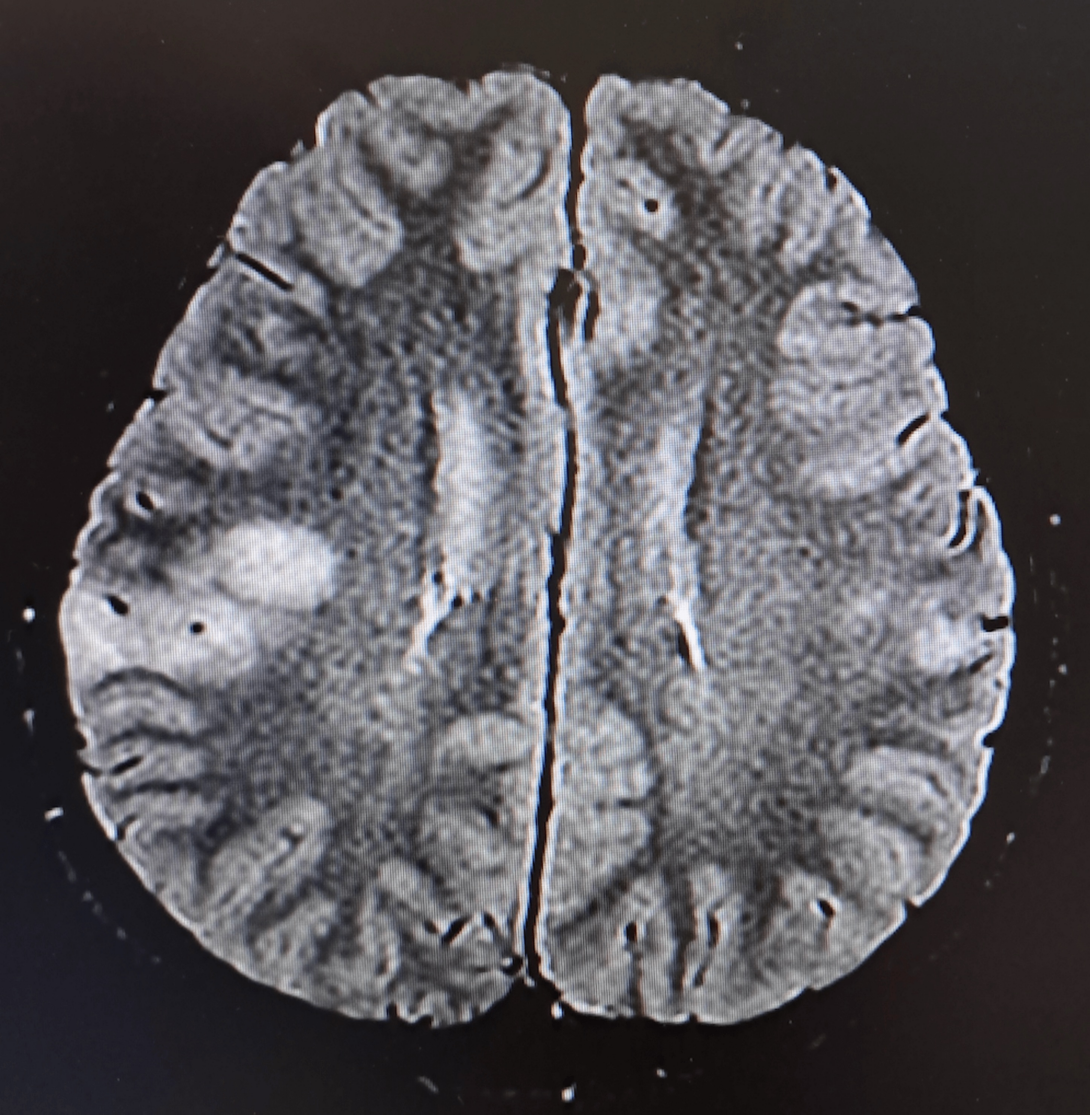
Axial T2 FLAIR MRI of the brain upon admission. It shows involvement of the right temporal and right high frontal gyri, with lesser involvement of the left high frontal gyrus.

The patient was admitted with a clinical suspicion of meningoencephalitis. On examination, he was drowsy but easily arousable, with mild neck rigidity. He was moving all four limbs without signs of lateralization and exhibited bilateral downgoing plantar reflexes (Babinski negative). An MRI of the brain was performed, revealing changes consistent with encephalitis (Figures 1, 2, 3, 4). Intravenous acyclovir at 12.5 mg/kg every 8 hours was initiated, along with ceftriaxone 2 g IV twice daily and levetiracetam 500 mg IV twice daily, while awaiting lumbar puncture results. Basic blood investigations were within normal ranges. Nasopharyngeal swab was positive for influenza A antigen test. Urinalysis and urine culture were negative. Blood glucose at the time of lumbar puncture was 6.4 mmol/L (reference range: 3.9-6.1 mmol/L). Lumbar puncture findings are shown in Table 1.

**Table 1:** CSF analysis of the patient on admission. WBC: white blood cell, RBC: red blood cell; CSF: cerebrospinal fluid

CSF analysis	Results	Normal value
Color	Colorless	
WBC	1785 mm^3^	0-3 mm^3^
RBC	2030 mm^3^	0-1 mm^3^
Lymphocyte	91%	
Neutrophils	9%	
Glucose	2.9 mmol/L	2.2-3.9 mmol/L
Protein	1650.2	150-450 mg/L
Gram stain/culture	Negative	

Infectious serology testing included HIV antibodies and p24 antigen, Hepatitis B surface antigen (HBsAg), Hepatitis C antigen (HCVAg), CMV IgM, and Brucella abortus antibodies, all of which were negative.

Given the markedly elevated white blood cell count, high protein, and low CSF-to-serum glucose ratio in the CSF analysis, bacterial co-infection remained a differential diagnosis that could not be excluded. This was due in part to the possibility that prior antibiotic administration interfered with CSF culture results. Therefore, vancomycin was administered for the next two days while awaiting CSF PCR results.

The meningitis and encephalitis PCR panel revealed definitive evidence of HSV-1 infection. After 10 days of admission, the patient's condition markedly improved: he was awake and alert, showed no meningeal signs, and had normal muscle strength, tone, sensation, and deep tendon reflexes. No further seizures occurred. He was discharged at his own request and will continue intravenous acyclovir for an additional 11 days to complete a 21-day course. No antibiotics were prescribed at discharge, as he had already completed a 10-day course during his admission.

## Discussion

Our patient’s CSF findings of severely elevated leukocytes, in the setting of a low CSF-to-blood glucose ratio and high protein, raise the possibility of bacterial co-infection in cases of preexisting HSV-1 viral encephalitis. The elevated lymphocyte count could also be due to partially treated bacterial meningoencephalitis. Despite our patient’s negative CSF culture and PCR panel showing only HSV-1, the differential diagnosis included other possible bacterial etiologies. The use of antibiotics prior to lumbar puncture, administered for more than 12 hours, could have affected the CSF/blood glucose ratio by elevating CSF glucose levels [5].

The incidence of presenting symptoms in HSV-1 encephalitis has been estimated as follows: fever (80%), confusion (72%), abnormal behavior (59%), decreased mental status (58%), seizures (54%), focal neurological deficits (41%), headache (58%), nausea and vomiting (40%), aphasia (40%), coma (33%), and meningismus (28%) [6].

The worldwide incidence of bacterial meningitis alone is estimated at around 20 cases per 100,000 people, with variable prevalence depending on geographical region [7]. Co-infection with bacterial meningitis is rare, with few reports of such cases in the literature and no established data on its incidence. Reactivation of HSV-1 has been theorized to occur secondary to immune dysfunction caused by severe bacterial infection [8]. Regarding bacterial meningitis co-infection with HSV-1 encephalitis, Ericsdotter et al. reported a case of Streptococcus meningitis in an elderly patient with recurrent otitis media who had HSV reactivation detected in CSF and oral samples [9]. Other cases include critically ill ICU patients with bronchoalveolar lavage positive for HSV-1 [10], which contrasts with our patient who is young, previously healthy, and not critically ill. Our patient’s CSF multiplex PCR panel was positive for HSV-1, with this test known to have 100% sensitivity for HSV-1 [11]. However, CSF cultures were negative for bacteria, which could be due to prior IV ceftriaxone doses before culture collection or other factors such as improper specimen handling or the presence of fastidious bacteria requiring longer growth times. Negative PCR results could also be explained by PCR inhibitors or the presence of pathogens not included in the PCR panel.

Regarding common bacterial meningitis pathogens such as Streptococcus pneumoniae and Haemophilus influenzae, studies have shown 100% detection rates with multiplex PCR. For others like Neisseria meningitidis, sensitivity is not well established [11].

Since our patient’s CSF culture and PCR results were negative for bacteria, we considered the possibility of infection with an uncommon CSF pathogen that is also susceptible to ceftriaxone.

Due to the limitations of CSF multiplex PCR coverage and sensitivity, recent research using DNA extraction and next-generation sequencing targeting 16S ribosomal RNA genes has detected diverse bacteria including Escherichia, Peptostreptococcus, Pseudomonas, Rothia, Acinetobacter, Prevotella, Bacillus, Neisseria, Catonella, Actinomyces, and Citrobacter in CSF specimens [12]. Multiplex lightmix real-time PCR can detect Streptococcus pneumoniae, Haemophilus influenzae, Neisseria meningitidis, Listeria monocytogenes, and Streptococcus agalactiae in CSF culture-negative cases [13].

One possible consideration was spontaneous non-Haemophilus gram-negative bacillary meningitis in this adult patient without trauma or neurological procedures. This is currently the third or fourth most common cause of nontraumatic bacterial meningitis after Streptococcus pneumoniae, Neisseria meningitidis, and Listeria monocytogenes. E. coli and Klebsiella pneumoniae are the most common community-acquired pathogens. However, this condition primarily affects the elderly or patients with underlying conditions such as alcoholism, diabetes, immunosuppression, malignancy, or cirrhosis. Our patient was young, previously healthy, with no alcohol use disorder or distant focus of infection, making spontaneous non-Haemophilus gram-negative bacillary meningitis less likely.

A viral co-infection of particular interest was influenza, detected via a positive influenza antigen test from the patient’s nasopharyngeal swab. Influenza infection can present with confusion and seizures, as seen initially in our patient. Although neurological complications from influenza are rare, influenza encephalitis should be considered when fever and neurological symptoms persist, especially during influenza outbreaks. Other causes of encephalitis should be excluded before attributing symptoms to influenza. Unfortunately, our patient did not undergo CSF PCR testing for influenza [14].

Other bacterial and viral co-infections reported in the literature include scrub typhus [15] and Zika virus [16], both diagnosed by specific CSF PCR tests. Immunocompromised patients are at risk for fungal meningitis, which can coexist with bacterial or, less commonly, viral infections. Cases of cryptococcal meningitis co-infection with HSV-1 have been described, such as in an HIV-positive middle-aged male not on antiviral therapy [17]. Our patient is immunocompetent, making fungal meningitis co-infection unlikely.

Zoonotic infections were also considered due to our patient’s occupational exposure to livestock, raising suspicion for zoonotic pathogens not covered by his antibiotic regimen. Reports exist of Brucella infections mimicking HSV encephalitis radiologically and in CSF analysis, with mild leukocytosis and normal protein [18]. No cases of HSV-1 and Brucella co-infection have been documented, though there is a report of Brucella-positive blood culture concurrent with acute varicella-zoster virus encephalitis [19].

Rickettsial infection was another differential; Baddal et al. described meningoencephalitis due to rickettsial co-infection with HSV-1 in a patient living in an area endemic for phlebotomus flies [20].

## Conclusions

This case highlights the diagnostic challenges of central nervous system (CNS) infections, particularly when antibiotics are administered prior to lumbar puncture. Although HSV-1 was confirmed via PCR, the CSF findings, characterized by elevated protein, leukocytosis, and a low CSF-to-blood glucose ratio despite normal CSF glucose, did not point to a single clear etiology and raised suspicion for possible bacterial co-infection. The presence of concurrent influenza A infection further complicated the clinical picture, emphasizing the potential for overlapping infections. Given the high mortality associated with co-infections, maintaining a broad differential diagnosis and initiating early empiric antimicrobial therapy are essential to improving outcomes.
